# Hepatitis C Virus Infection Influences the *S*-Methadone Metabolite Plasma Concentration

**DOI:** 10.1371/journal.pone.0069310

**Published:** 2013-07-23

**Authors:** Shiow-Ling Wu, Sheng-Chang Wang, Hsiao-Hui Tsou, Hsiang-Wei Kuo, Ing-Kang Ho, Sheng-Wen Liu, Ya-Ting Hsu, Yao-Sheng Chang, Yu-Li Liu

**Affiliations:** 1 Graduate Institute of Life Sciences, National Defense Medical Center, Taipei, Taiwan; 2 Center for Research and Diagnostics, Centers for Disease Control, Department of Health, Executive Yuan, Taipei, Taiwan; 3 Center for Neuropsychiatric Research, National Health Research Institutes, Zhunan, Miaoli County, Taiwan; 4 Division of Biostatistics and Bioinformatics, Institute of Population Health Sciences, National Health Research Institutes, Zhunan, Miaoli County, Taiwan; 5 Center for Drug Abuse and Addiction, China Medical University Hospital, Taichung, Taiwan; 6 Graduate Institute of Clinical Medical Science, College of Medicine, China Medical University, Taichung, Taiwan; 7 Graduate Institute of Drug Safety, China Medical University, Taichung, Taiwan; 8 Department of Psychiatry, National Taiwan University Hospital and National Taiwan University College of Medicine, Taipei, Taiwan; University of Sydney, Australia

## Abstract

**Background and Objectives:**

Heroin-dependent patients typically contract hepatitis C virus (HCV) at a disproportionately high level due to needle exchange. The liver is the primary target organ of HCV infection and also the main organ responsible for drug metabolism. Methadone maintenance treatment (MMT) is a major treatment regimen for opioid dependence. HCV infection may affect methadone metabolism but this has rarely been studied. In our current study, we aimed to test the hypothesis that HCV may influence the methadone dosage and its plasma metabolite concentrations in a MMT cohort from Taiwan.

**Methods:**

A total of 366 MMT patients were recruited. The levels of plasma hepatitis B virus (HBV), HCV, human immunodeficiency virus (HIV) antibodies (Ab), liver aspartate aminotransferase (AST) and alanine aminotransferase (ALT), as well as methadone and its metabolite 2-ethylidene-1,5-dimethyl-3,3-diphenylpyrrolidine (EDDP) were measured along with the urine morphine concentration and amphetamine screening.

**Results:**

Of the 352 subjects in our cohort with HCV test records, 95% were found to be positive for plasma anti-HCV antibody. The liver functional parameters of AST (Wilcoxon Rank-Sum test, *P* = 0.02) and ALT (Wilcoxon Rank-Sum test, *P* = 0.04), the plasma methadone concentrations (Wilcoxon Rank-Sum test, *P* = 0.043) and the *R*-enantiomer of methadone concentrations (Wilcoxon Rank-Sum test, *P* = 0.032) were significantly higher in the HCV antibody-positive subjects than in the HCV antibody-negative patients, but not the *S*-EDDP/methadone dose ratio. The HCV levels correlated with the methadone dose (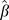 = 14.65 and 14.13; *P* = 0.029 and 0.03) and the *S*-EDDP/methadone dose ratio (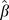 = −0.41 and −0.40; *P* = 0.00084 and 0.002) in both univariate and multivariate regression analyses.

**Conclusions:**

We conclude that HCV may influence the methadone dose and plasma *S*-EDDP/methadone dose ratio in MMT patients in this preliminary study.

## Introduction

Methadone is a synthetic opioid that is generally used as a replacement therapy to counteract withdrawal symptoms in heroin-dependent patients [1]. Higher incidence rates of hepatitis C virus (HCV) infection have been reported in these patients who are under methadone treatment [2], [3]. Epidemics of blood borne infectious diseases in heroin-injecting users has aroused worldwide public health concerns with HCV infection emerging as one of the most serious problems among these epidemics [4]. Compared with a prevalence of 0.3 to 14.5% in the general population, HCV infection has an incidence of 50% to 95% among drug needle users [5]–[7]. Patients infected with HCV experienced a series of hepatic tissue damages including acute hepatitis, chronic inflammation, fibrosis and cirrhosis [8]. Furthermore, the risk of hepatocellular carcinoma is extraordinarily high among these individuals [9]. As the liver is the key metabolic organ, it is critical to evaluate the extent to which HCV infection would influence its metabolic functions. This knowledge will assist clinicians to adjust the medications when treating affected patients.

Methadone contains a chiral center that allows the compound to produce *R*-form and *S*-form enantiomers [10]. Methadone is extensively metabolized in the liver through specific isoforms of the cytochrome P-450 enzyme system [11]. It has been reported that methadone is metabolized by CYP2B6, CYP2C19, CYP3A4 and, to a lesser extent, by CYP2D6 [11]–[13]. The CYP isozymes have preferential metabolic differences between the methadone enantiomers, with CYP2C19 preferring to metabolize the *R*-form and CYP2B6 the *S*-form [11]. The effects of HCV on methadone metabolism remain poorly understood; it has been reported however that cirrhosis and late-stage liver failure may interfere with methadone metabolism [14]. MMT patients may require high methadone dosages to maintain an adequate blood concentration [14]. In our present study, we hypothesized that the HCV serostatus would determine the methadone metabolic profiles by influencing CYP2B6 genetic function. First, we examined the association of the HCV serostatus with the methadone metabolic profiles from a cohort of MMT patients. We then examined the impact of the HCV serostatus on CYP2B6 gene expression.

## Materials and Methods

### Subjects

The study protocol was approved by the institutional review boards of the National Health Research Institutes (Zhunan, Taiwan) and the six participating hospitals of Tao-Yuan Mental Hospital, En-Chu-Kong Hospital, Far-Eastern Memorial Hospital, Taipei City Hospital Song-De and Yang-Ming Branches, China Medical University Hospital, and Wei-Gong Memorial Hospital. Written informed consents were obtained from all participants. The project was registered with the National Institutes of Health Clinical Trial database (http://www.clinicaltrial.gov/ct/show/NCT01059747). A total of 366 Han Chinese subjects with heroin dependence undergoing MMT in outpatient settings were recruited from year 2008 to 2009. The inclusion criteria included an age of 18 or above, undergoing MMT for at least three months with regular attendance for the past seven days, and a lower than 10 mg methadone dosage adjustment during the past seven days. Exclusion criteria included pregnancy and co-morbidity with physical and mental disorders requiring immediate treatment.

### Clinical Assessments

Demographics, substance use histories and methadone treatment courses, including the dose and treatment duration, and the treatment compliance over the previous week, were obtained from the medical records. Interviewer-administered assessments, including a Treatment Outcomes Profile (TOP) [15], an 11 item clinical opioid withdrawal scale (COWS) [16], and a Treatment Emergent Symptoms Scale (TESS) [17] for adverse events related to methadone treatment, were conducted by research nurses before the next methadone dose intake. The higher the COWS or the TESS scores, the more severe the withdrawal symptoms or side effects reported by patients.

### Serum and Urine Drug Testing

Antibodies against HBV, HCV and HIV and the levels of aspartate aminotransferase (AST, reference range: <38 U/L), alanine aminotransferase (ALT, reference range: <41 U/L) and gamma-glutamyl transpeptidase (γ-GT, reference range: 8–61 U/L) from serum samples of patients were measured at the Taipei Institute of Pathology (Taipei, Taiwan). Urine specimens were collected prior to the administration of methadone on the study day. The morphine screen test was performed via a kinetic interaction of microparticles (KIMS) on an Integra 800 device (Roche Diagnostics, Basel, Switzerland). In our present analyses and previous reports [18]–[20], the urine morphine test was used as a surrogate measurement for the methadone treatment outcome.

### CYP2B6 Metabolized EDDP

The metabolism of 2-ethylidene-1,5-dimethyl-3,3-diphenylpyrrolidine (EDDP) through the CYP2B6 enzyme was assayed *in vitro*. CYP2B6 baculovirus-infected insect cell-expressed supersomes (10 µl; total protein concentration of 15 mg/ml equal to 1 nmol/ml) (BD Gentest, Woburn, MA), 10 µl of regenerating system (3.3 mM glucose-6-phosphate, and 0.3 U/ml glucose-6-phosphate dehydrogenase), and 30 µl of reaction buffer (100 mM phosphate buffer, pH 8.0) were incubated with 40 µl of 62.5 µM EDDP and pre-incubated for 20 min at room temperature in a 96-well polystyrene plate (NUNC, Roskilde, Denmark). The reaction was started by adding 10 µl of NADP+ (10 mM) and incubated at 37°C for 24 h. The reaction was stopped by adding 100 µl of mobile phase. A 100 µl of mixture was loaded onto a solid-phase extraction column via the procedure detailed below. A 50 µl aliquot was then chromatographed by HPLC.

### Analyses of Methadone and its Metabolites in the Plasma

Plasma concentrations of methadone and its metabolite EDDP enantiomers were measured using HPLC. The methodology is described in our previous report [21]. Briefly, methadone, EDDP, and amitriptyline as an internal standard (40 ng), were extracted from the plasma samples using a C18-E 100 mg/ml capacity Strata Solid Phase Extraction Column (Phenomenex, Torrance, CA). Following the conditioning of the column on a vacuum manifold (Waters, Milford, MA), 800 µl aliquots of each plasma sample and 40 ng of the amitriptyline internal standard were added. The column was then washed and the retained compounds were eluted with 1 ml of ammonium phosphate (monobasic)/methanol (0.01 g/100 ml). The collected eluent was then evaporated and the remaining residue was dissolved in 100 µl of the mobile phase. A total sample volume of 50 µl was then chromatographed. The intra-day and inter-day coefficients of variation (CV) were 3.3% and 6.6% for R-methadone, 2.5% and 5.6% for *S*-methadone, 1.6% and 3.9% for *R*-EDDP, and 2.8% and 5.5% for *S*-EDDP, respectively. The recovery rates for *R*-methadone, *S*-methadone, *R*-EDDP and *S*-EDDP were 109.0±7.6%, 96.7±8.6%, 96.6±6.6% and 87.4±3.2%, respectively. The recovery rate for the internal standard was 60.2±4.8%.

### CYP2B6 Real-time Polymerase Chain Reaction

For CYP2B6 expression measurements, we selected the 9 HCV-Ab-negative and 11 HCV-Ab- positive patients out of our 366 subjects. The selection process considered gender and an age match of ±2 years old. CYP2B6 expression was assessed using the lymphoblastoid cell lines transformed from patients’ lymphocytes by the Epstein-Barr virus (EBV). These transformed lymphoblastoid cells were washed once with ice-cold phosphate buffered saline before total RNA extraction. Trizol Reagent (Life Technologies, Carlsbad, CA) was used according to the manufacturer’s guidelines to extract total RNA.

RT-PCR amplification was conducted using RevertAid™ H minus a first strand cDNA synthesis kit (Fermentas, Waltham, MA) with a random hexamer and real-time PCR on an ABI StepOne Plus System, in accordance with the manufacturer’s instructions. Real-time PCR was performed for CYP2B6 and a housekeeping gene, TATA-box binding protein (TBP), using pre-designed gene-specific TaqMan® probes and primer sets (Hs03044634_m1 for CYP2B6 and Hs00920497_m1 for TBP) purchased from Applied Biosystems (Applied Biosystems, Foster City, CA). Gene expression was quantified relative to *TBP* expression using ABI StepOne Plus Software and the relative quantification method. The relative expression level of CYP2B6 compared with that of TBP was defined as −ΔCT = −[CT_CYP2B6_−CT_TBP_], where CT is the cycle threshold. The CYP2B6 mRNA/TBP mRNA ratio was calculated from 2^−ΔCT^.

### Statistics

All statistical analyses were conducted using SAS software, Version 9.3 (SAS Institute, Inc., Cary, NC). The clinical variables compared between the HCV antibody-positive and HCV antibody-negative patients, were calculated using the non-parametric Wilcoxon rank-sum test for continuous variables andχ^2^ test or Fisher's exact Test for categorical variables. Data for continuous variables are presented as the mean ± standard deviation (SD). Permutation analyses were performed to adjust for multiple comparisons. Clinical variables with a *P*-value less than 0.1 in the univariate regression were considered to be potential predictors of dependent variables and were further explored in multivariate analyses [22]. The univariate regression model was used to determine the potential predictors of dependent variables. Multivariate regression was further used to relate individual dependent variable (such as the methadone dose, or the *S*-EDDP/methadone dose ratio) to multiple independent variables (including the HCV serostatus, age, BMI, AST, or ALT). The variance inflation factor (VIF) was used to identify the multicollinearity between all covariates. A VIF of less than 10 indicated no multicollinearity among the covariates. The denominator varied due to different numbers of subjects across different laboratory tests. The relative expression level of CYP2B6 between the HCV antibody-positive and HCV antibody-negative patients was compared via the unpaired Student’s t-test using GraphPAD prism, version 5 (GraphPad Software, Inc., La Jolla, CA). The normal distribution in each group was confirmed by the Shapiro-Wilk test. Statistical significance was designated at *P*-values less than 0.05.

## Results

### General Demographics and Clinical Characteristics

**Figure 1:**
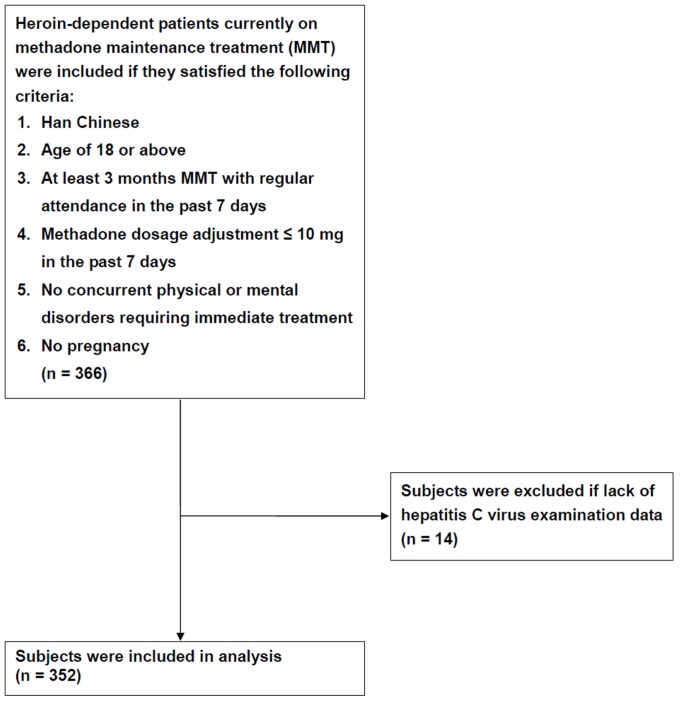
Flow diagram of the study recruitment procedure for MMT patients.

A total of 366 MMT patients were analyzed in this study; 352 of these patients had been screened for HCV antibody (Figure 1), and 334 (95%) were positive (Table 1). The average age for the entire cohort was 38.2±7.7 years, among which the average age was 38.3±7.8 years in the HCV antibody-positive and 37.2±6.4 years in the HCV antibody-negative patients. The AST (Wilcoxon rank-sum test, *P* = 0.022) and ALT (Wilcoxon rank-sum test, *P* = 0.04) levels were significantly higher in the HCV antibody-positive than the HCV antibody-negative patients. There were no significant differences between the HCV antibody-positive and HCV antibody-negative patients in gender, body mass index (BMI), methadone dose, methadone treatment duration, cigarette smoking of nicotine metabolite cotinine, liver function parameter of γ-GT, urine morphine and amphetamine test, human immunodeficiency virus (HIV) test, hepatitis B surface antigen (HBV sAg) test, or hepatitis B surface antibody (HBV sAb). Among 359 HIV test patients, 86 patients (24%) were HIV antibody-positive. There were only 83 HIV antibody-positive patients among the 352 patients screened for HCV.

**Table 1:** Demographics and laboratory findings for MMT study subjects, classified by viral hepatitis C virus infection.

	HCV (+)		HCV(−)		
	n	(%) or Mean ± SD	n	(%) or Mean ± SD	*P*-value
Age (years)^†^	334	38.34±7.81	18	37.17±6.35	0.731^w^
Male	270	(80.84%)	17	(94.44%)	0.215^f^
BMI (kgw/m^2^)	331	23.61±3.54	18	22.66±2.54	0.276^w^
Methadone dose (mg/day)	334	55.20±27.78	18	40.56±25.26	0.052^w^
Treatment duration (week)^‡^	333	64.57±39.24	18	56.31±28.48	0.501^w^
**Liver Function**					
AST(U/L)	320	53.66±57.73	18	31.44±11.72	**0.022** w
ALT(U/L)	326	62.90±76.68	18	37.06±27.94	**0.040** w
γ-GT(U/L)	306	61.96±98.24	18	70.44±165.85	0.331^w^
**Laboratory Tests**					
Urine morphine (+)	173	(52.27%)	7	(38.89%)	0.269^c^
HBVs Ab (+)	135	(57.94%)	9	(64.29%)	0.640^c^

n, subject number; SD, standard deviation; BMI, body mass index; HCV (+), hepatitis C virus antibody-positive; HCV (−), hepatitis C virus antibody-negative; HBVs Ab, hepatitis B surface antibody;

w Wilcoxon rank-sum test.

c Chi-square test.

f Fisher's exact test.

Using the AST/ALT ratio as a criterion for the severity of liver damage or cirrhosis [23] in HCV antibody-positive patients, the proportion of men (Fisher's exact test, *P* = 0.024) and BMI of these patients (Wilcoxon rank-sum test, *P* = 0.003) were significantly higher in the AST/ALT <1 group (61% male, and 24±3 BMI) than in the AST/ALT ≥1 group (39% male, and 23±3 BMI). However, the percentage of HIV antibody-positive patients was higher in the AST/ALT ≥1 group (60% HIV) than in the AST/ALT <1 group (40% HIV) (Fisher's exact test, *P*<0.001).

### HCV Influences the Plasma Concentrations of Methadone and its Metabolites

Methadone and its metabolite EDDP were measured in the 352 MMT patients in our cohort that were screened for HCV. The total plasma methadone concentrations (*R*- and *S*-methadone; Wilcoxon rank-sum test, *P* = 0.043), plasma *R*-methadone concentrations (R-methadone; Wilcoxon rank-sum test, *P* = 0.032), and the ratio of *S*-EDDP/methadone dose (Wilcoxon rank-sum test, *P* = 0.044) were significantly different between the HCV-Ab-positive and HCV-Ab-negative patients (Table 2). The average total plasma methadone concentration and *R*-methadone concentrations were higher in the HCV antibody-positive (340±209 ng/ml and 196±122 ng/ml) than in the HCV antibody-negative (261±204 ng/ml and 142±99 ng/ml) patients. However, the average ratios of *S*-EDDP/methadone dose were significantly lower in the HCV antibody-positive (0.31±0.35) than in the HCV antibody-negative (0.72±1.66) patients.

**Table 2:** Methadone and its metabolism between the HCV antibody-positive and antibody-negative MMT subjects.

	HCV(+)		HCV(−)			Permutation*P*-value
Variable	n	Mean ± SD	n	Mean ± SD	*P*-value	
(*R*,*S*)-Methadone(ng/ml)	334	339.8±208.8	18	260.9±204.4	**0.043**	0.116
(*R*,*S*)-EDDP(ng/ml)	324	28.73±23.94	17	27.55±19.67	0.961	0.837
*R*-Methadone (ng/ml)	334	195.9±122.1	18	141.9±98.6	**0.032**	0.058
*R*-EDDP(ng/ml)	325	13.84±15.56	17	11.1±6.75	0.891	0.405
*R*-Methadone/(*R*,*S*)-Methadone Ratio	334	0.59±0.08	18	0.58±0.11	0.409	0.591
*R*-EDDP/(*R*,*S*)-Methadone Ratio	325	0.06±0.12	17	0.05±0.03	0.235	0.793
*R*-Methadone/Methadone Dose ratio	334	3.86±2.34	18	3.73±1.13	0.559	0.795
*R*-EDDP/Methadone Dose ratio	325	0.31±0.52	17	0.32±0.28	0.143	0.877
*S*-Methadone (ng/ml)	334	143.9±97.4	18	119±109.7	0.08	0.298
*S*-EDDP (ng/ml)	331	14.6±13.03	17	16.45±15.82	0.68	0.534
*S*-Methadone/(*R*,*S*)-Methadone ratio	334	0.41±0.08	18	0.42±0.11	0.409	0.593
*S*-EDDP/(*R*,*S*)-Methadone ratio	331	0.05±0.07	17	0.09±0.15	0.064	0.069
*S*-Methadone/Methadone Dose ratio	334	2.78±1.56	18	3.06±1.68	0.370	0.449
*S*-EDDP/Methadone Dose ratio	331	0.31±0.35	17	0.72±1.66	**0.044**	**0.016**

N, subject number; SD, standard deviation; HCV (+), hepatitis C virus antibody-positive;

HCV (−), hepatitis C virus antibody-negative.

*P*-value: Wilcoxon Rank-Sum Test, Permutation *P*-value: permutation test.

### *S*-EDDP/methadone Dose Ratio and the Methadone Dose are Associated with HCV Infection

Using univariate regression analyses, the methadone dose (*P* = 0.029) and the *S*-EDDP/methadone dose ratios (*P*<0.001) showed significant correlations with HCV tests (Table S1). In further multivariate regression analyses, the methadone dose and ratio of *S*-EDDP/methadone dose continued to be significantly predicted by the HCV tests after adjusting for age, BMI, AST, and ALT (*P* = 0.03 and 0.002) (Table 3). MMT patients with a HCV infection received a 14.13 mg higher methadone dose, but had a lower *S*-EDDP/methadone dose ratio (0.4 less on average) compared with the patients without HCV infection.

**Table 3:** Multivariate regression analyses of the methadone dose and *S*-EDDP/methadone dose ratio.

	Methadone dose (mg/day) (n = 335)				*S*-EDDP/Methadone dose Ratio (n = 331)			
	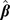	SE	*P*-value	VIF	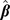	SE	*P*-value	VIF
HCV Ab (+/−)	14.13	6.49	**0.030**	1.01	−0.40	0.13	**0.002**	1.01
Age (years)	−0.79	0.20	**0.0001**	1.05	0.002	0.004	0.609	1.06
BMI (kgw/m^2^)	0.88	0.42	**0.036**	1.02	−0.002	0.008	0.817	1.02
AST(U/L)	0.098	0.052	0.061	4.14	−0.0006	0.001	0.532	4.13
ALT(U/L)	−0.071	0.039	0.067	4.05	0.0003	0.001	0.719	4.05

HCV Ab, hepatitis C virus antibody; 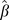, regression coefficient; SE, standard error of regression coefficient; VIF, variance inflation factor.

### CYP2B6 had Higher Expression in HCV Antibody-positive Patients

**Figure 2:**
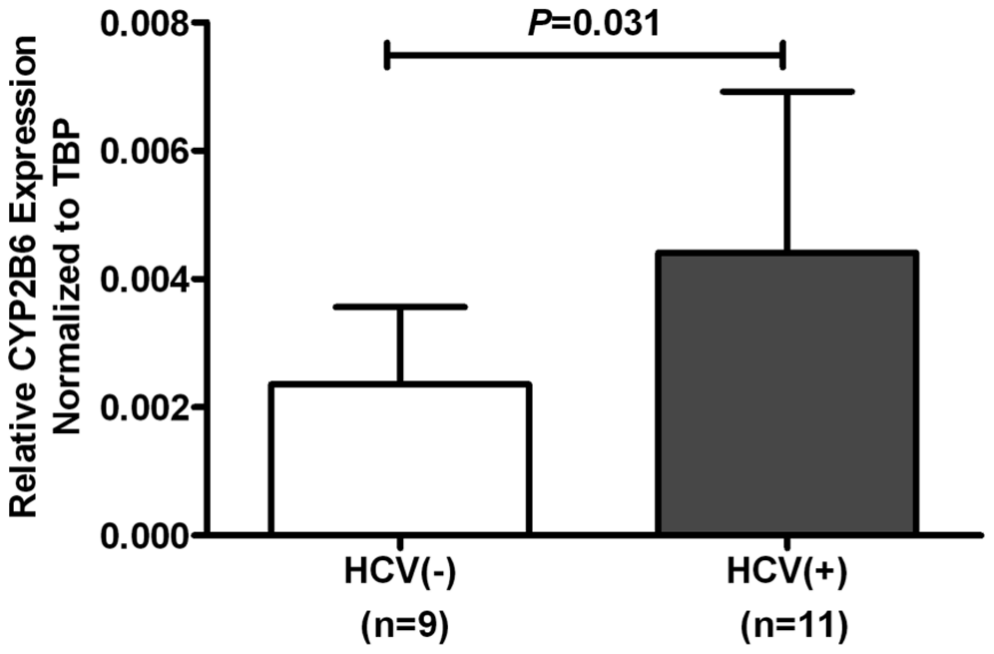
The relative CYP2B6 expression levels in EBV-transformed lymphoblastoids were compared between HCV antibody-positive (HCV(+)) and antibody-negative patients (HCV(−)). (The error bar represents the standard deviation; n, subject number).

Using quantitative real-time PCR analyses the relative expression levels of the CYP2B6 gene were found to be significantly (*P* = 0.031) higher in the EBV-transformed lymphoblastoids of HCV antibody-positive patients compared with HCV antibody-negative cases matched for *S*-EDDP/dose ratio, urine morphine test, BMI, gender and age (Figure 2). In further analyses to determine the catalytic activity of CYP2B6 for methadone metabolites, this enzyme was found to metabolize both *R* and *S*-EDDP forms (Figure S1).

## Discussion

HCV infection has a high incidence in MMT patients [3] and little is known on how infection with this virus might affect methadone metabolism. In our current study, we compared the plasma concentrations of methadone and its metabolite EDDP, and also their concentration-to-dose ratio. Our results show that the plasma methadone concentration, plasma *R*-methadone concentration and the *S*-EDDP/methadone dose ratio differ significantly between HCV antibody-positive and the HCV antibody-negative MMT patients. In further univariate regression analyses, the ratio of *S*-EDDP/methadone dose demonstrated the most significant correlation with HCV infection. When we used multivariate regression analyses, the *S*-EDDP/methadone dose ratio continued to show a significant correlation with HCV infection. A higher ratio indicated fewer HCV antibody-positive patients. This ratio was lower in HCV antibody-positive patients than in HCV antibody-negative patients. As *S*-methadone is preferentially metabolized by CYP2B6 [19], [21], we further examined in our current study the relative expression levels of CYP2B6 between the HCV antibody-positive and HCV antibody-negative patients. This relative expression was found to be higher in the HCV antibody-positive patients, and the CYP2B6 enzyme was further shown to metabolize both *S*-methadone, and its *R*- and *S*-EDDP metabolite enantiomers. This was a plausible explanation for the lower *S*-EDDP/methadone dose ratio in the HCV antibody-positive patients.

Clinically, it is noteworthy from our current findings that HCV infection may influence the methadone treatment dose, in addition to methadone-induced opioid cross tolerance observed previously [24]. Methadone is a racemic mixture with unique characteristics in comprising an *R*- and *S*-methadone enantiomer. *R*-methadone (or l-isomer) is a 10-fold more potent agonist of the μ-opioid receptor [25] and has a 50-fold higher analgesic potency [26] than *S*-methadone. *R*-methadone thus seems to be the major stereoisomer involved in pain relief and the prevention of opioid withdrawal. *S*-methadone (or *d*-isomer) is an antagonist of the NMDA receptor [27] and also inhibits the reuptake of 5-hydroxytryptamine [28]. *S*-methadone blocks the human ether-à-go-go-related gene (hERG) voltage-gated potassium channel more potently, which can cause drug-induced long QT syndrome, leading to potentially lethal ventricular tachyarrhythmia [29]. The HCV antibody-positive patients in our current study cohort had a lower *S*-EDDP/methadone ratio, but higher plasma *R*-methadone concentration and might therefore benefit from *R*-methadone induced withdrawal prevention. This partly explains the high percentage of HCV antibody-positive patients in the MMT programs in Taiwan and in Asia [30], [31] in which HCV causes an increase in *S*-methadone elimination and a reduction in adverse reactions. Our current study is the first to identify that HCV infection may influence methadone metabolism, particularly *S*-methadone metabolism. Our findings suggest that methadone dosage adjustment should be considered in HCV-infected patients in an MMT program.

HCV infection may affect the liver functional enzymes of AST and ALT, but not γ-GT. In our present experiments, we also found that HCV antibody-positive MMT patients had higher AST and ALT levels than HCV antibody-negative patients. The liver is the primary target of HCV infection and the extent of damage to hepatocytes has been found to closely correlate with serum markers such as, AST and ALT [32]–[35]. HCV infection has also been reported to be associated with the release of AST, ALT, and γ-GT [35]–[37]. This may be the reason why AST and ALT were found to be significantly increased in the HCV antibody-positive patients in our current cohort. The γ-GT levels are mainly correlated with alcohol use [38], [39]. There were no differences found in our current analyses in the percentage of alcohol users between the HCV antibody-positive and HCV antibody-negative patient groups. This may be the reason why γ-GT failed to show a higher serum level in these MMT patients. When further subgrouping the severity of the liver’s infectious status among the HCV antibody-positive patients, it was found that the AST/ALT ≥1 group of MMT patients had a lower BMI, but a higher percentage of combined HIV infection than the AST/ALT<1 group. Using AST/ALT ≥1 as an indicator for liver cirrhosis has had both supportive [40], [41] and non-supportive reports [42]. While the predictive accuracy of AST/ALT ≥1 is still in question [43], it may be an useful indicator for combined HIV infection among HCV antibody-positive MMT patients.

Although it is not clear that how HCV infection alters methadone metabolism, HCV- associated hepatic inflammation has been associated with a reduction in methadone dosage requirements [44]. In our current study, our multivariate regression data also indicated that the methadone dose correlates with HCV infection (Table 3) although the reason for this correlation remains unclear. We propose that the increase in CYP2B6 enzymatic activity may be one of the reasons.

The limitations of our present study include the fact that 95% of the MMT patients in our cohort were HCV antibody-positive. This may create a statistical imbalance, however a high HCV incidence is common in heroin-dependent-patients with reported ranges from 67% to 96% [3]. HCV antibody-positivity combined with HBV presence has been reported to cause severe damage to liver tissue [45], [46]. In our present study, this combined infection status did not affect methadone metabolism. This may be because only three of our patients had a combined HCV antibody-negative and HBV antigen presence. In addition, the relative expression levels of CYP2B6 were obtained from EBV-transformed lymphoblastoids, and not from the infected liver tissue of MMT patients. This limited our ability to obtain direct evidence and left open the possibility that the EBV-transformed lymphoblastoids had a preference for enhanced CYP2B6 expression after interaction with HCV virus. Further testing of actual liver CYP2B6 expression is therefore warranted.

In summary, we found from our current analysis that MMT patients with HCV infection may have a higher level of AST and ALT production in the serum. The γ-GT levels were unaffected by HCV. However, MMT patients with HCV infection have a higher plasma concentration of total methadone and *R*-methadone, but a lower *S*-EDDP/methadone dose ratio. In univariate analyses, the methadone dose and the *S*-EDDP/methadone dose ratio were found to have a significant correlation with HCV infection. In further multivariate correlation analyses, the *S*-EDDP/methadone dose ratio was shown to be the major correlate with HCV infection. In further CYP2B6 expression analyses, we found that the CYP2B6 enzyme had a higher expression in the HCV antibody-positive group of MMT patients. Because CYP2B6 metabolizes both *S*-methadone and *R*- and *S*-EDDP, this may be why the *S*-EDDP/methadone dose ratio is lower in the HCV antibody-positive patients.

## Supporting Information

Figure S1 The catalytic activity of CYP2B6 against EDDP. A HPLC chromatogram of the EDDP peak area was compared between the presence (+) and the absence (−) of CYP2B6 enzyme. (The error bar represents the standard deviation)
(TIF)

Table S1 Univariate regression analyses of P-values for methadone dose, plasma methadone and its metabolites.
(DOC)
