# Supplementary figures and images for: Hepatitis C Virus Infection Influences the S-Methadone Metabolite Plasma Concentration
Source: PLoS One. 2013 Jul 23;8(7):e69310. doi: 10.1371/journal.pone.0069310 (PMC3720619; doi:10.1371/journal.pone.0069310)

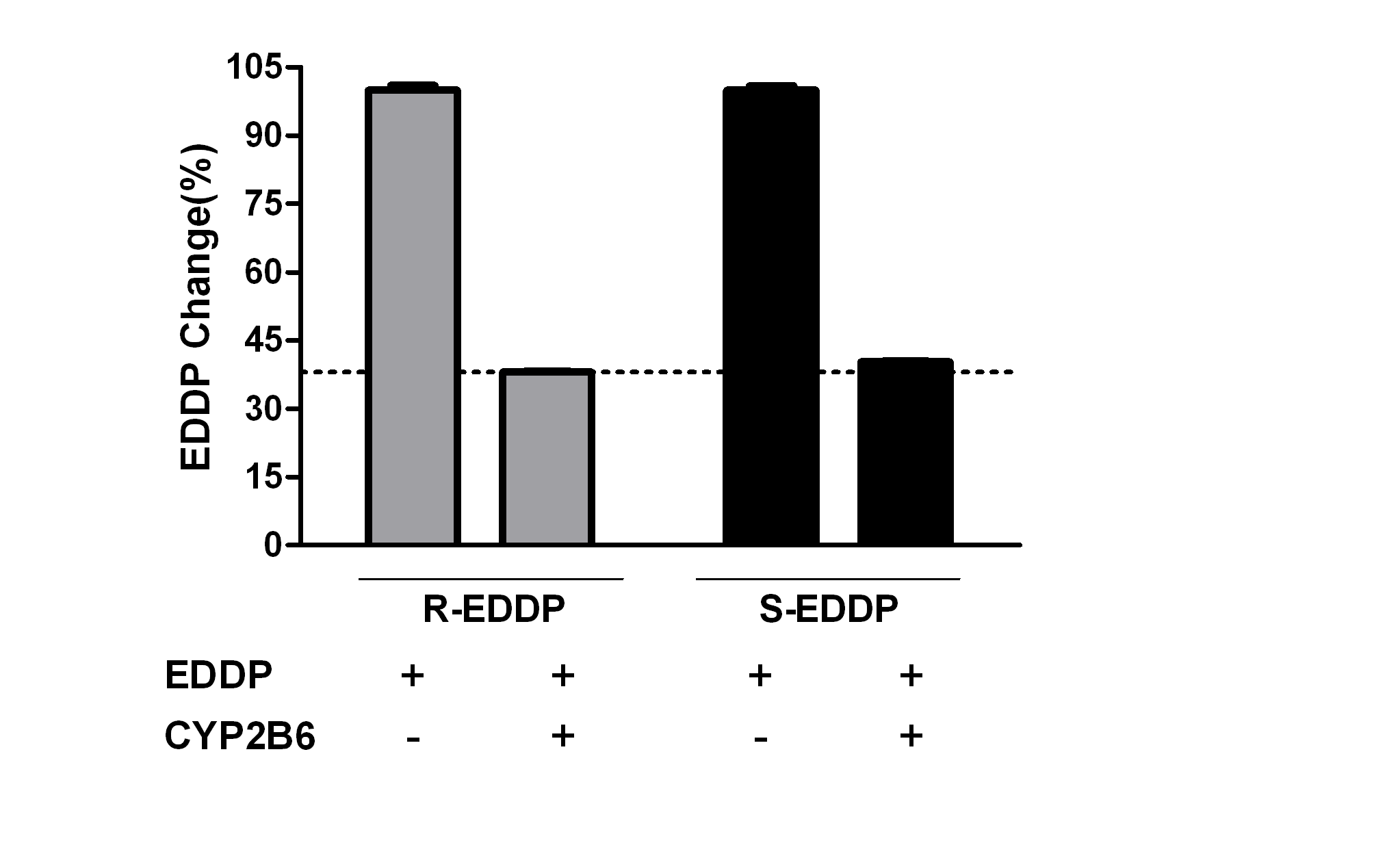

Supplement: Figure S1 — The catalytic activity of CYP2B6 against EDDP. A HPLC chromatogram of the EDDP peak area was compared between the presence (+) and the absence (−) of CYP2B6 enzyme. (The error bar represents the standard deviation) (TIF) [file pone.0069310.s001.tif]
