# Supplementary material for: Hepatitis C Virus Infection Influences the S-Methadone Metabolite Plasma Concentration
Source: PLoS One. 2013 Jul 23;8(7):e69310. doi: 10.1371/journal.pone.0069310 (PMC3720619; doi:10.1371/journal.pone.0069310)
Supplement: Table S1 — Univariate regression analyses of P-values for methadone dose, plasma methadone and its metabolites. (DOC) [file pone.0069310.s002.doc]

Table S1. Univariate regression analyses of *P*-values for methadone dose, plasma methadone and its metabolites

| Variables | (R,S)-Methadone (ng/ml) | R-Methadone  (ng/ml) | S-Methadone  (ng/ml) | S-EDDP/(R,S)-Methadone ratio | Methadone Dose  (mg/day) | S-EDDP/Methadone Dose ratio |
| --- | --- | --- | --- | --- | --- | --- |
| Age (years) | **1.4E-03** | **9.1E-04** | **6.9E-03** | 0.609 | **2.3E-05** | 0.659 |
| Male | 0.757 | 0.951 | 0.561 | 0.609 | 0.941 | 0.498 |
| BMI (kgw x m^2^) | **3.2E-04** | **7.3E-04** | **3.7E-04** | 0.326 | **6.0E-03** | 0.780 |
| Methadone Dosage (mg/day) | **<1.0E-14** | **<1.0E-14** | **<1.0E-14** | **1.1E-02** | - | **3.0E-06** |
| Duration (week) | 0.486 | 0.383 | 0.683 | 0.643 | 0.449 | 0.400 |
| Nicotine metabolite Cotinine (ng/ml) | **3.9E-02** | 0.156 | **8.1E-03** | 0.358 | 0.203 | 0.947 |
| **Substance Use History** |  |  |  |  |  |  |
| Alcohol User (+/-) | 0.798 | 0.670 | 0.985 | 0.116 | 0.434 | 0.347 |
| Amphetamine User (+/-) | 0.895 | 0.944 | 0.846 | 0.865 | 0.966 | 0.385 |
| MDMA User (+/-) | 0.302 | 0.262 | 0.414 | 0.841 | 0.006 | 0.834 |
| Ketamine User (+/-) | 0.696 | 0.486 | 0.979 | 0.643 | 0.364 | 0.318 |
| **Liver function** |  |  |  |  |  |  |
| AST(U/L) | 0.276 | 0.479 | 0.148 | 0.392 | 0.869 | 0.404 |
| ALT(U/L) | 0.764 | 0.647 | 0.941 | 0.455 | 0.565 | 0.608 |
| γ-GT(U/L) | 0.186 | 0.543 | **4.1E-02** | 0.794 | 0.695 | 0.981 |
| **Urine and Blood Tests** |  |  |  |  |  |  |
| Morphine (+/-) | **2.4E-02** | 0.068 | **1.1E-02** | 0.319 | 0.790 | 0.655 |
| Amphetamine (+/-) | 0.758 | 0.539 | 0.920 | 0.766 | 0.419 | 0.366 |
| HIV (+/-) | 0.147 | 0.056 | 0.472 | 0.805 | 0.109 | 0.577 |
| TPHA (+/-) | 0.469 | 0.462 | 0.525 | 0.948 | 0.604 | 0.949 |
| HBVs Ag (+/-) | 0.833 | 0.856 | 0.823 | 0.547 | 0.448 | 0.897 |
| HBVs Ab (+/-) | 0.916 | 0.698 | 0.531 | 0.670 | 0.053 | 0.648 |
| HCV Ab (+/-) | 0.119 | 0.066 | 0.295 | 0.072 | **2.9E-02** | **8.6E-04** |

BMI, body mass index, HIV, human immunodeficiency virus; HBVs Ag, hepatitis B surface antigen; HBVs Ab, hepatitis B surface antibody; HCV Ab, hepatitis C virus antibody.
